# Supplementary material for: Temporal and spatial assembly of inner ear hair cell ankle link condensate through phase separation
Source: Nat Commun. 2023 Mar 24;14:1657. doi: 10.1038/s41467-023-37267-5 (PMC10039067; doi:10.1038/s41467-023-37267-5)
Supplement: Supplementary file 1 — Supplementary Information [file 41467_2023_37267_MOESM1_ESM.pdf]

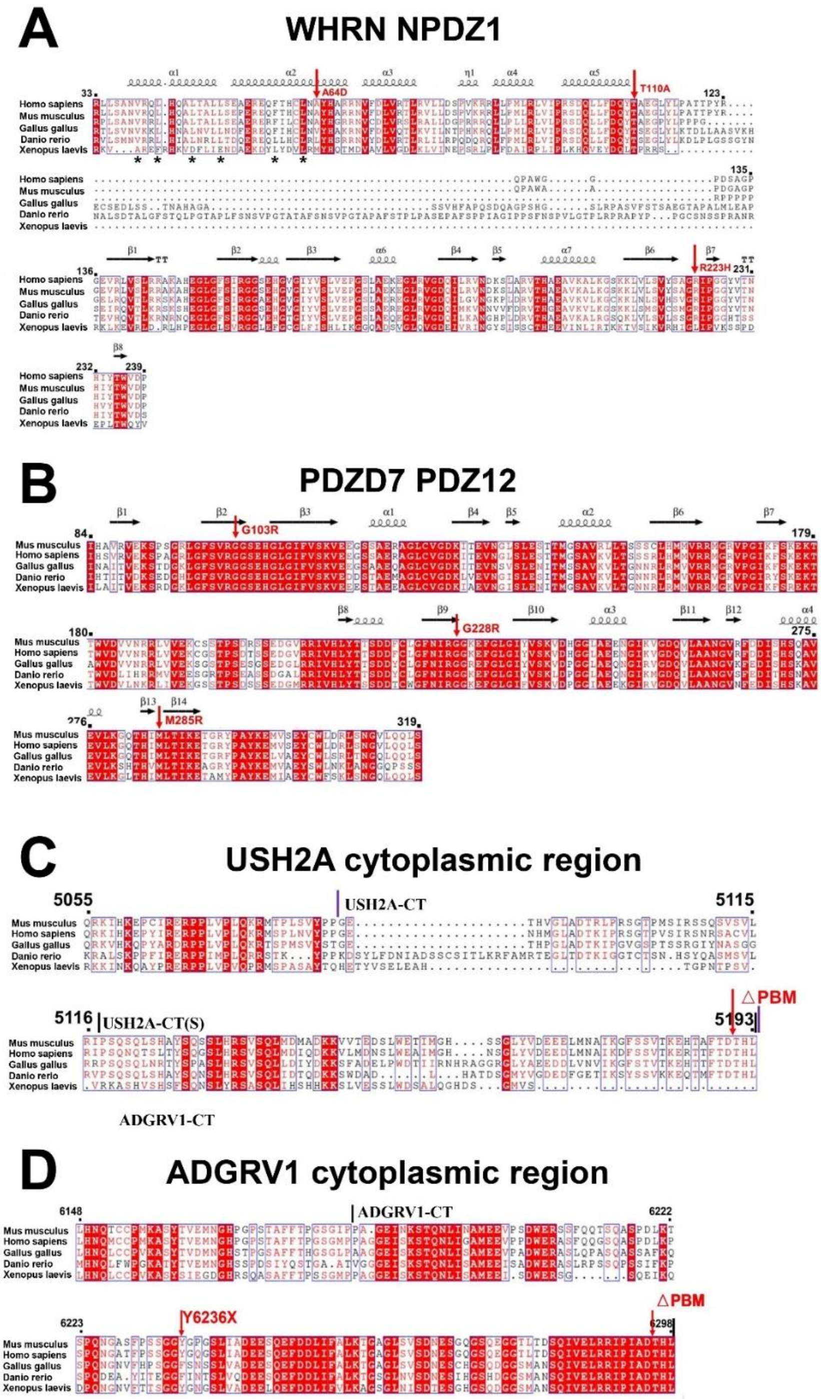

Supplementary Fig. 1. Sequence alignments of USH2 proteins. Related to Fig. 6, 8, 9, and 10. Sequence alignments among different species of **(a)** WHRN NPDZ1, **(b)** PDZD7 PDZ12, and the cytoplasmic regions of **(c)** USH2A and **(d)** ADGRV1. The identical residues are highlighted with crimson, and the other conserved residues are colored in light red. Disease mutations and deletions are indicated with red arrows. The residues critical for forming the hydrophobic cleft in WHRN NTD are indicated with black asterisks.

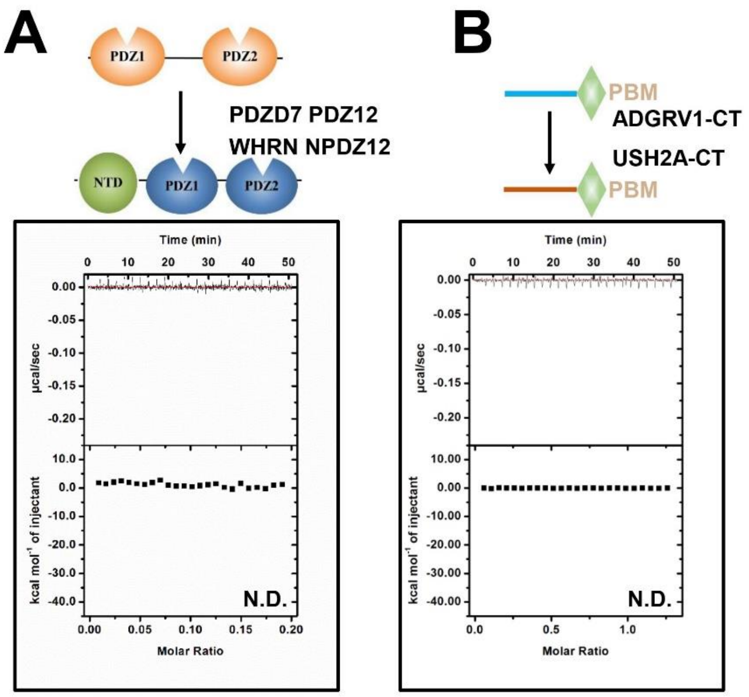

Supplementary Fig. S2. Related to Fig. 2. **Binding affinities between PDZD7 PDZ12 and WHRN NPDZ12 (a), ADGRV1-CT and USH2A-CT (b) determined by ITC.**

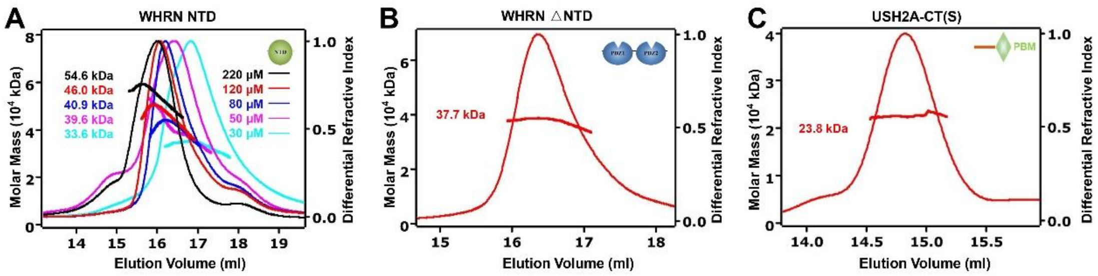

Supplementary Fig. S3. The molecular sizes of WHRN NTD, WHRN △NTD, and USH2A-CT(S) Related to Fig. 5. **a.** SEC-MALS showing that WHRN NTD underwent concentration-dependent oligomerization. **b-c.** SEC-MALS showing that WHRN △NTD (B), and USH2A-CT(S) (C) were both monomer. WHRN NPDZ12 is abbreviated as WHRN. △NTD, the truncation of NTD. NTD, N-terminal domain.

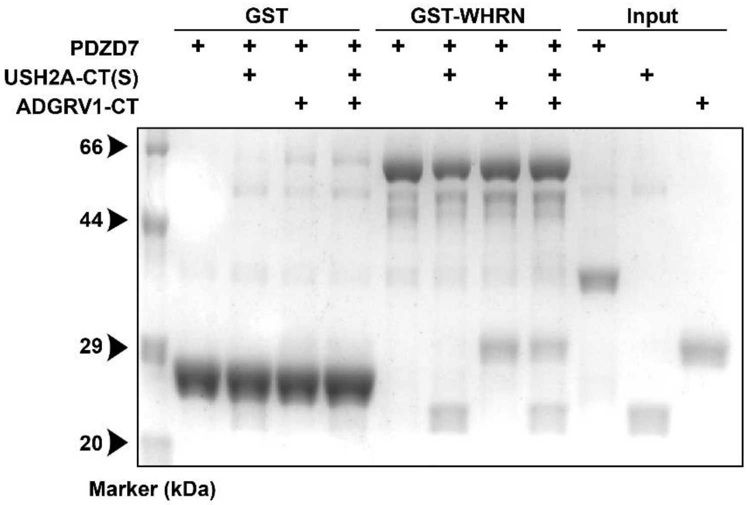

Supplementary Fig. S4. WHRN directly interacts with USH2A-CT(S) and ADGRV1-CT but not PDZD7. Related to Fig. 4. GST pull-down assay showing that GST-WHRN pulled down USH2A-CT(S) and ADGRV1-CT but not PDZD7. WHRN NPDZ12 and PDZD7 PDZ12 are abbreviated as WHRN and PDZD7, respectively.

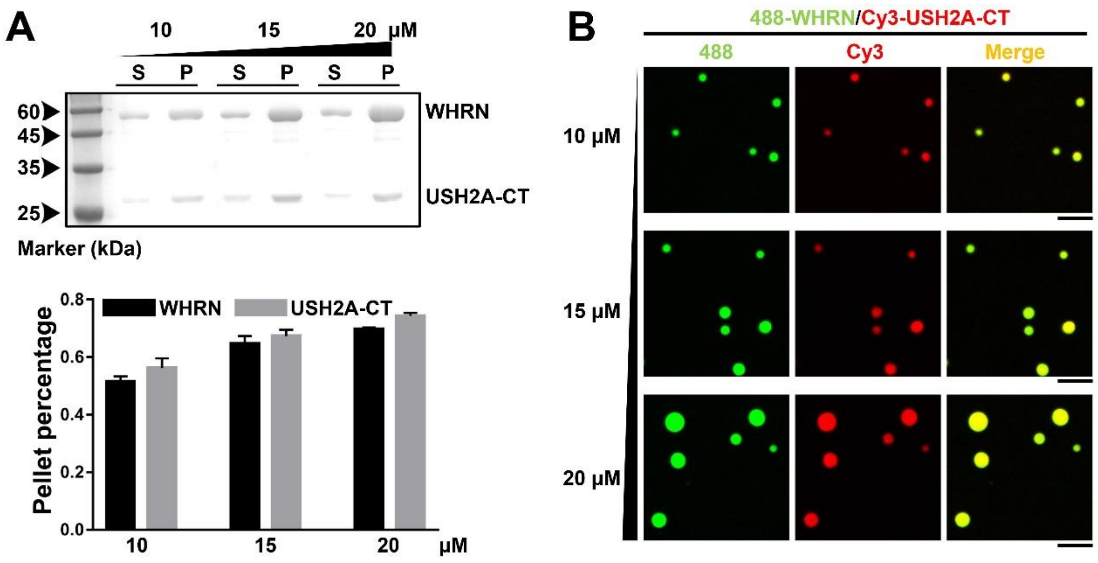

Supplementary Fig. S5. WHRN/USH2A-CT phase separation is concentration-dependent. Related to Fig. 5. **a.** Up panel, Co-sedimentation-based assay showing that the pellet enrichments of the two proteins in WHRN/USH2A-CT increased concentration-dependently; Bottom panel, quantifications of the percentage of WHRN or USH2A-CT recovered from the condensed phase (pellet) in the co-sedimentation assay described in up panel. WHRN and USH2A-CT were mixed at a 1:1 molar ratio with final concentrations indicated. Mean ± SD, n=3. **b.** Fluorescence images showing that the droplets formed by WHRN/USH2A-CT concentration-dependently increased in size or number. WHRN and USH2A-CT were mixed at a 1:1 molar ratio with final concentrations indicated. Scale bar: 5 µm. WHRN NPDZ12 is abbreviated as WHRN. Source data are provided as a Source Data file.

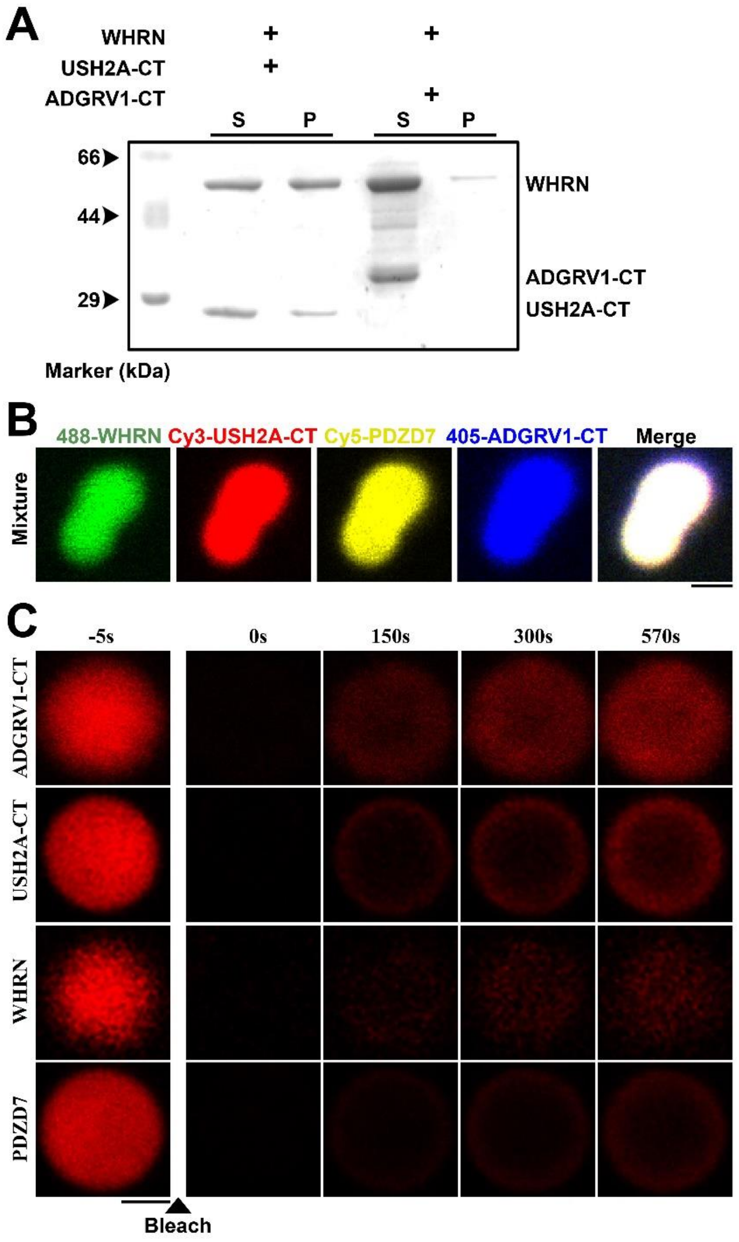

Supplementary Fig. S6. Related to Fig. 5. **The dynamic nature of quaternary USH2 protein condensate. a.** Co-sedimentation-based assay showing that proteins were enriched in pellet in WHRN/USH2A-CT but not WHRN/ADGRV1-CT. **b.** The small droplets fused into larger ones. Scale bar: 1 µm. **c.** The original movie of the FRAP analysis was described in Fig. 5h. Scale bar: 1 µm. WHRN NPDZ12 and PDZD7 PDZ12 are abbreviated as WHRN and PDZD7, respectively. Source data are provided as a Source Data file.

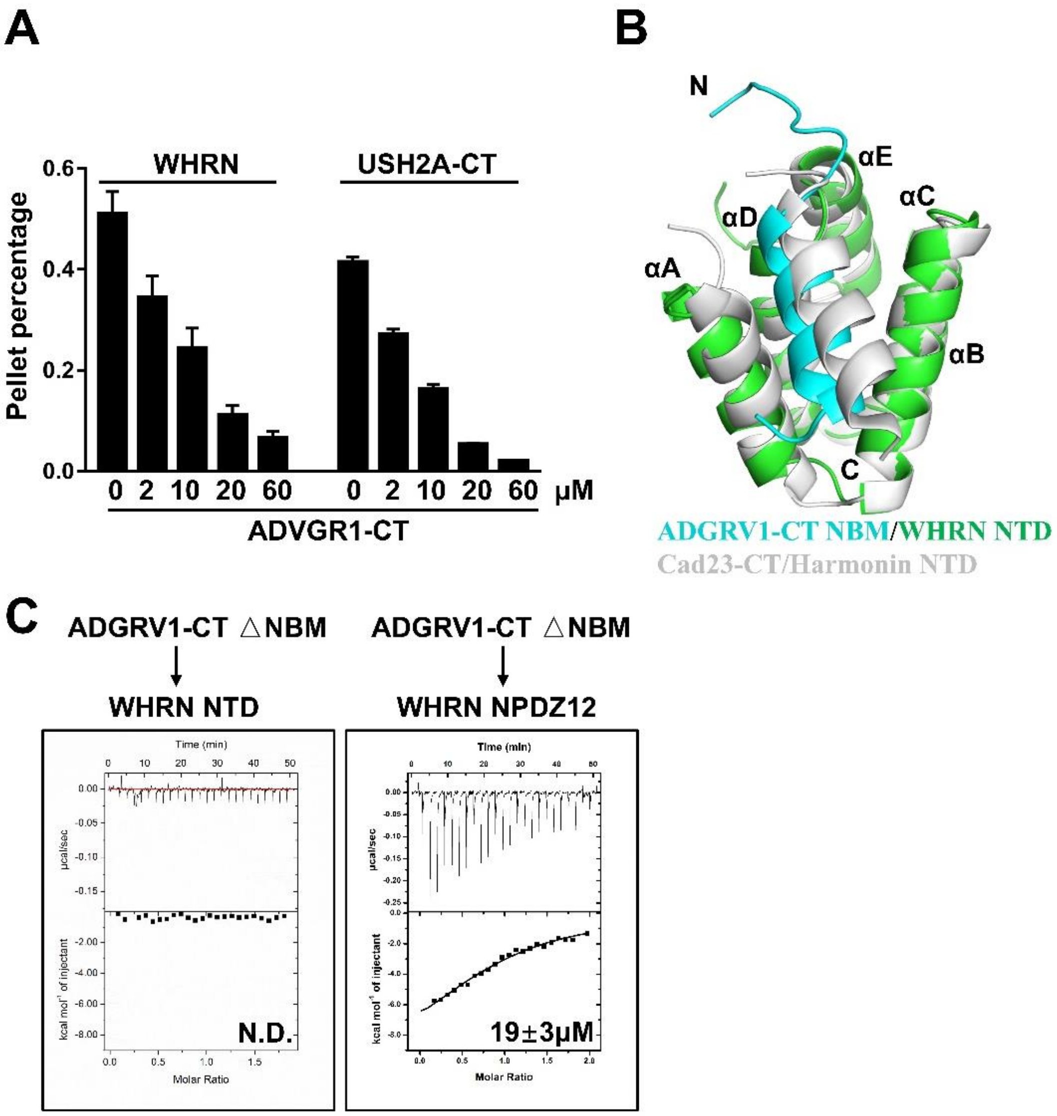

		ITC		Kd (μM)
ADGRV1-CT	WT	⟶	WHRN NTD	31 ± 27
	△NBM			N.D.
ADGRV1-CT	WT	⟶	WHRN NPDZ12	1.4 ± 0.3
	△NBM			19 ± 3

Supplementary Fig. S7. Related to Fig. 6. **The interaction between ADGRV1-CT NBM and WHRN NTD. a.** Quantifications of the percentage of WHRN NPDZ12 or USH2A-CT recovered from the condensed phase (pellet) in the co-sedimentation assays described in Fig. 6a. Mean ± SD, n=3. **b.** The superimposition of our predicted WHRN-NTD/ADGRV1-CT complex structure with the resolved structure of Cad23 NBM/Harmonin NTD shows a similar bind mode with the hydrophobic cleft on NTD occupied by NBM. **c.** Up panel, ITC assays showing that truncating NBM (△NBM) from ADGRV1-CT abolished and diminished its bindings to WHRN NTD and WHRN NPDZ12, respectively; bottom panel, summaries of the binding affinities between WT or △NBM constructs of ADGRV1-CT and NTD or NPDZ12 of WHRN. NBM, NTD binding motif. NTD, N-terminal domain.

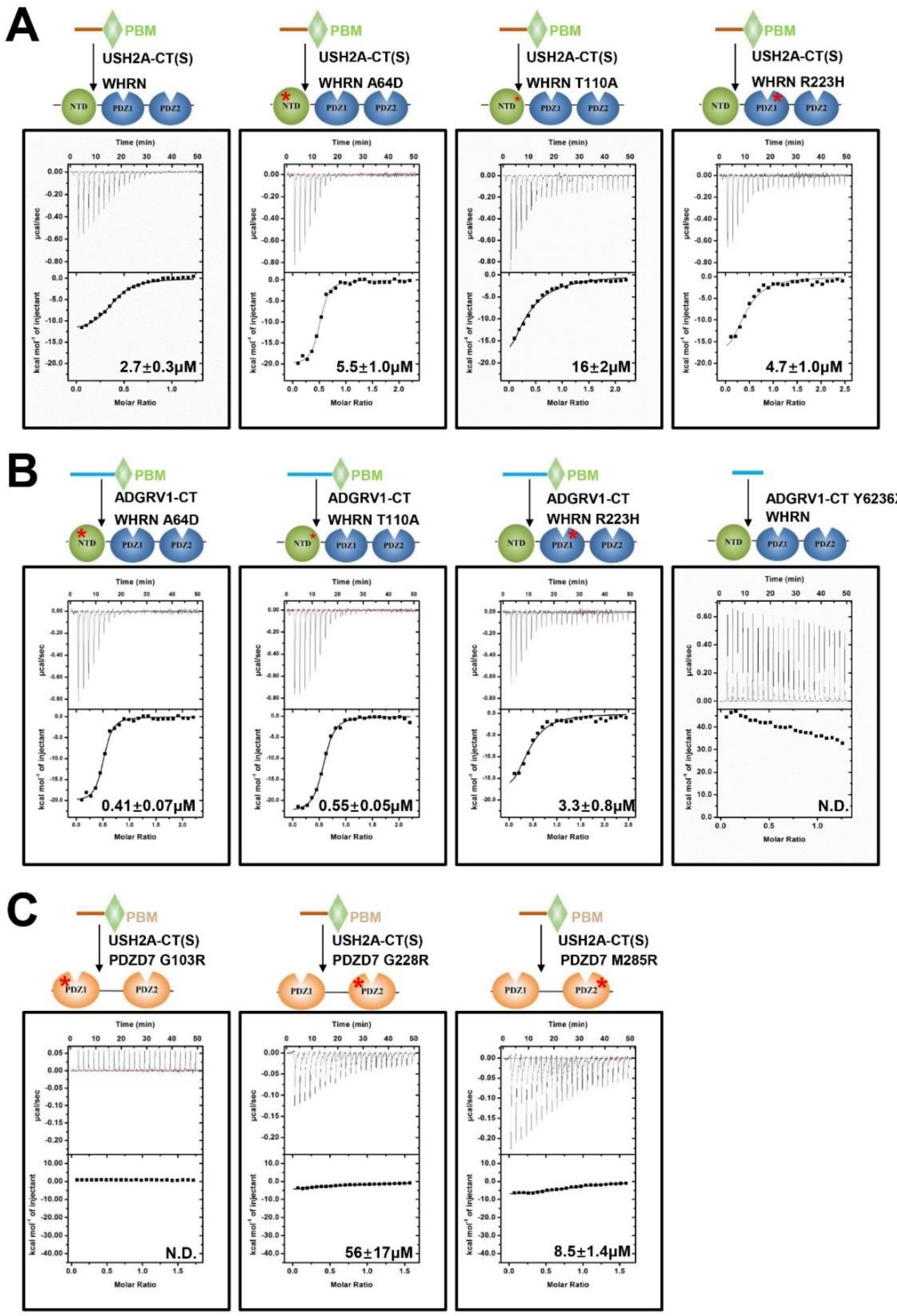

Supplementary Fig. S8. The effects of deafness-associated mutations on the binding affinities between USH2 proteins. Related to Fig. 8, 9 and 10. **a.** Binding affinities of USH2A-CT(S) to WT or deafness-related mutants (A64D, T110A, or R223H) of WHRN determined by ITC. **b.** Binding affinities of ADGRV1-CT to deafness-related mutants (A64D, T110A, or R223H) of WHRN determined by ITC. And Y6236X abolished ADGRV1-CT's binding to WHRN. **c.** Binding affinities of USH2A-CT(S) to deafness-related mutants (G103R, G228R, or M285R) of PDZD7 determined by ITC. N.D., not detectable. WHRN NPDZ12 and PDZD7 PDZ12 are abbreviated as WHRN and PDZD7, respectively.
