# Supplementary material for: Temporal and spatial assembly of inner ear hair cell ankle link condensate through phase separation
Source: Nat Commun. 2023 Mar 24;14:1657. doi: 10.1038/s41467-023-37267-5 (PMC10039067; doi:10.1038/s41467-023-37267-5)
Supplement: Supplementary file 3 — Reporting Summary [file 41467_2023_37267_MOESM3_ESM.pdf]

nature portfolio

Corresponding author(s): Zhigang Xu, Qing Lu

Last updated by author(s): Feb 25, 2023

Reporting Summary

Nature Portfolio wishes to improve the reproducibility of the work that we publish. This form provides structure for consistency and transparency in reporting. For further information on Nature Portfolio policies, see our Editorial Policies and the Editorial Policy Checklist.

Statistics

For all statistical analyses, confirm that the following items are present in the figure legend, table legend, main text, or Methods section.

n/a	Confirmed	
[ ]	[x]	The exact sample size (*n*) for each experimental group/condition, given as a discrete number and unit of measurement
[ ]	[x]	A statement on whether measurements were taken from distinct samples or whether the same sample was measured repeatedly
[ ]	[x]	The statistical test(s) used AND whether they are one- or two-sided<br>*Only common tests should be described solely by name; describe more complex techniques in the Methods section.*
[x]	[ ]	A description of all covariates tested
[x]	[ ]	A description of any assumptions or corrections, such as tests of normality and adjustment for multiple comparisons
[ ]	[x]	A full description of the statistical parameters including central tendency (e.g. means) or other basic estimates (e.g. regression coefficient) AND variation (e.g. standard deviation) or associated estimates of uncertainty (e.g. confidence intervals)
[ ]	[x]	For null hypothesis testing, the test statistic (e.g. *F*, *t*, *r*) with confidence intervals, effect sizes, degrees of freedom and *P* value noted<br>*Give P values as exact values whenever suitable.*
[x]	[ ]	For Bayesian analysis, information on the choice of priors and Markov chain Monte Carlo settings
[x]	[ ]	For hierarchical and complex designs, identification of the appropriate level for tests and full reporting of outcomes
[x]	[ ]	Estimates of effect sizes (e.g. Cohen's *d*, Pearson's *r*), indicating how they were calculated

Our web collection on statistics for biologists contains articles on many of the points above.

Software and code

Policy information about availability of computer code

Data collection	Unicorn 7.0 for collecting protein purification chromatography data<br>ITC200 for collecting ITC data<br>ASTRA 6 for collecting static light scattering data<br>LAS X for collecting imaging data
Data analysis	Graphpad Prism 5.0 for statistical analyze and figure plotting<br>ASTRA 6 for analyzing static light scattering data<br>MicroCal Analysis Launcher for analyzing ITC data<br>LAS X for analyzing imaging data

For manuscripts utilizing custom algorithms or software that are central to the research but not yet described in published literature, software must be made available to editors and reviewers. We strongly encourage code deposition in a community repository (e.g. GitHub). See the Nature Portfolio guidelines for submitting code & software for further information.

Data

Policy information about availability of data

All manuscripts must include a data availability statement. This statement should provide the following information, where applicable:

- Accession codes, unique identifiers, or web links for publicly available datasets
- A description of any restrictions on data availability
- For clinical datasets or third party data, please ensure that the statement adheres to our policy

The authors declare that all data supporting the findings of this study are available within the article and its Supplementary Information files or from the corresponding author upon reasonable request. Source data are provided with this paper. The resolved complex structure of Cad23 NBM/Harmonin NTD or Harmonin NPDZ1/SANS SAM-PBM is deposited in PDB with the accession code of 2KBR [http://doi.org/10.2210/pdb2KBR/pdb] or 3K1R [http://doi.org/10.2210/pdb3K1R/pdb], respectively.

Human research participants

Policy information about studies involving human research participants and Sex and Gender in Research.

Reporting on sex and gender	This is not relevant to our study
Population characteristics	This is not relevant to our study
Recruitment	This is not relevant to our study
Ethics oversight	This is not relevant to our study

Note that full information on the approval of the study protocol must also be provided in the manuscript.

Field-specific reporting

Please select the one below that is the best fit for your research. If you are not sure, read the appropriate sections before making your selection.

[x] Life sciences [ ] Behavioural & social sciences [ ] Ecological, evolutionary & environmental sciences

For a reference copy of the document with all sections, see nature.com/documents/nr-reporting-summary-flat.pdf

Life sciences study design

All studies must disclose on these points even when the disclosure is negative.

Sample size	We performed at least 3 biological replicates with multiple technical replicates for some co-sedimentation and FRAP assays (as stated in figure legends).
Data exclusions	No data were excluded from the analyses
Replication	As mentioned with the relevant figures, sufficient technical and biological replicates of the experiments were performed and the data is reproducible using the methods and models described by the authors.
Randomization	Samples were allocated randomly into experimental groups.
Blinding	No blinding was performed in the study. All data were acquired and analyzed automatically using the software described above.

Reporting for specific materials, systems and methods

We require information from authors about some types of materials, experimental systems and methods used in many studies. Here, indicate whether each material, system or method listed is relevant to your study. If you are not sure if a list item applies to your research, read the appropriate section before selecting a response.

Materials & experimental systems

n/a	Involved in the study
☐	☒ Antibodies
☒	☐ Eukaryotic cell lines
☒	☐ Palaeontology and archaeology
☐	☒ Animals and other organisms
☒	☐ Clinical data
☒	☐ Dual use research of concern

Methods

n/a	Involved in the study
☒	☐ ChIP-seq
☒	☐ Flow cytometry
☒	☐ MRI-based neuroimaging

Antibodies

Antibodies used	Whirlin-specific antibody (1:4000 dilution) was affinity-purified from antisera or egg yolk extracts, and VLGR1-specific antibody (1:4000 dilution) was affinity-purified from mouse. And Alexa Fluor 594 donkey anti-rabbit secondary antibody (Thermo Fisher Scientific, Cat. No. R37119, 1:400 dilution) was used in this study.
Validation	The antibodies against Whirlin and VLGR1 were validated by Jun Yang and his colleagues (PMID: 26401052)

Animals and other research organisms

Policy information about studies involving animals; ARRIVE guidelines recommended for reporting animal research, and Sex and Gender in Research

Laboratory animals	4-day-old C57BL/6 mice were taken as donors of cochlear sensory epithelia
Wild animals	The study did not involve wild animals
Reporting on sex	This is not relevant to our study
Field-collected samples	The study did not involve samples collected from the field
Ethics oversight	All animal experiments were approved by the Animal Ethics Committee of Shandong University School of Life Sciences (Permit Number: SYDWLL-2017-05) and performed accordingly.

Note that full information on the approval of the study protocol must also be provided in the manuscript.
