# Supplementary figures and images for: Temporal and spatial assembly of inner ear hair cell ankle link condensate through phase separation
Source: Nat Commun. 2023 Mar 24;14:1657. doi: 10.1038/s41467-023-37267-5 (PMC10039067; doi:10.1038/s41467-023-37267-5)

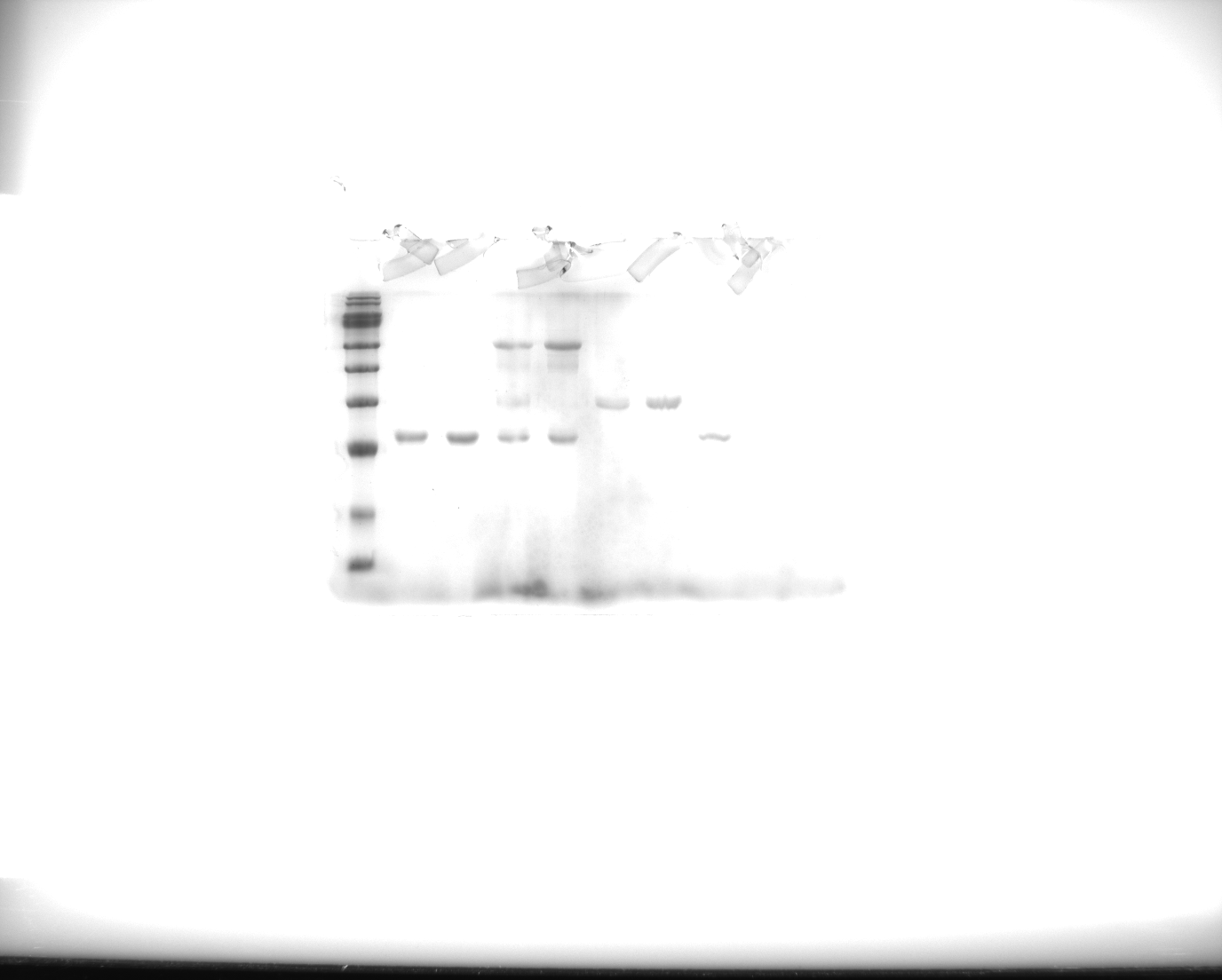

Supplement: Supplementary file 4 — Source data [file 41467_2023_37267_MOESM4_ESM.zip › source data/FIG10C.Tif]

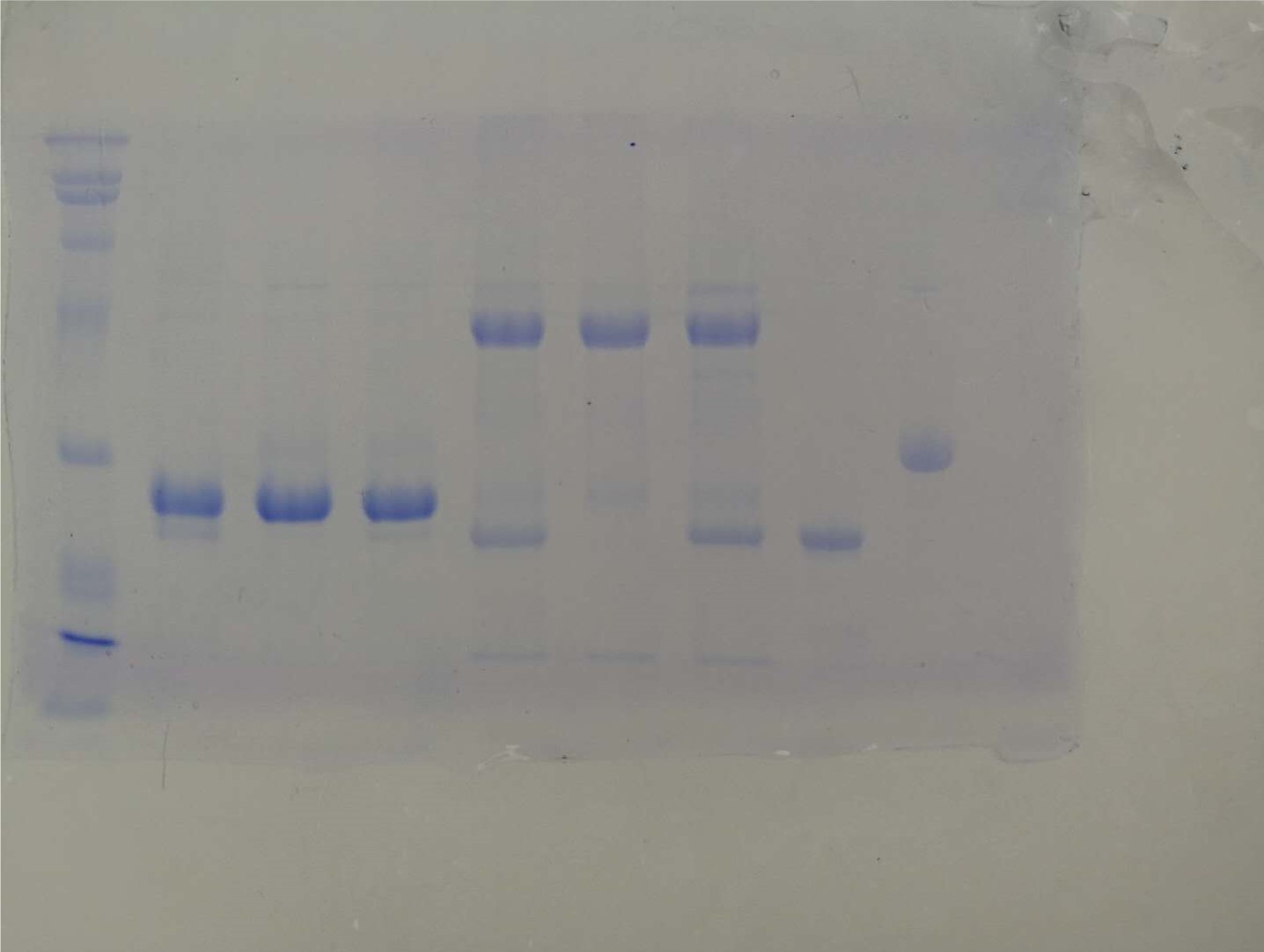

Supplement: Supplementary file 4 — Source data [file 41467_2023_37267_MOESM4_ESM.zip › source data/FIG2E.jpg]

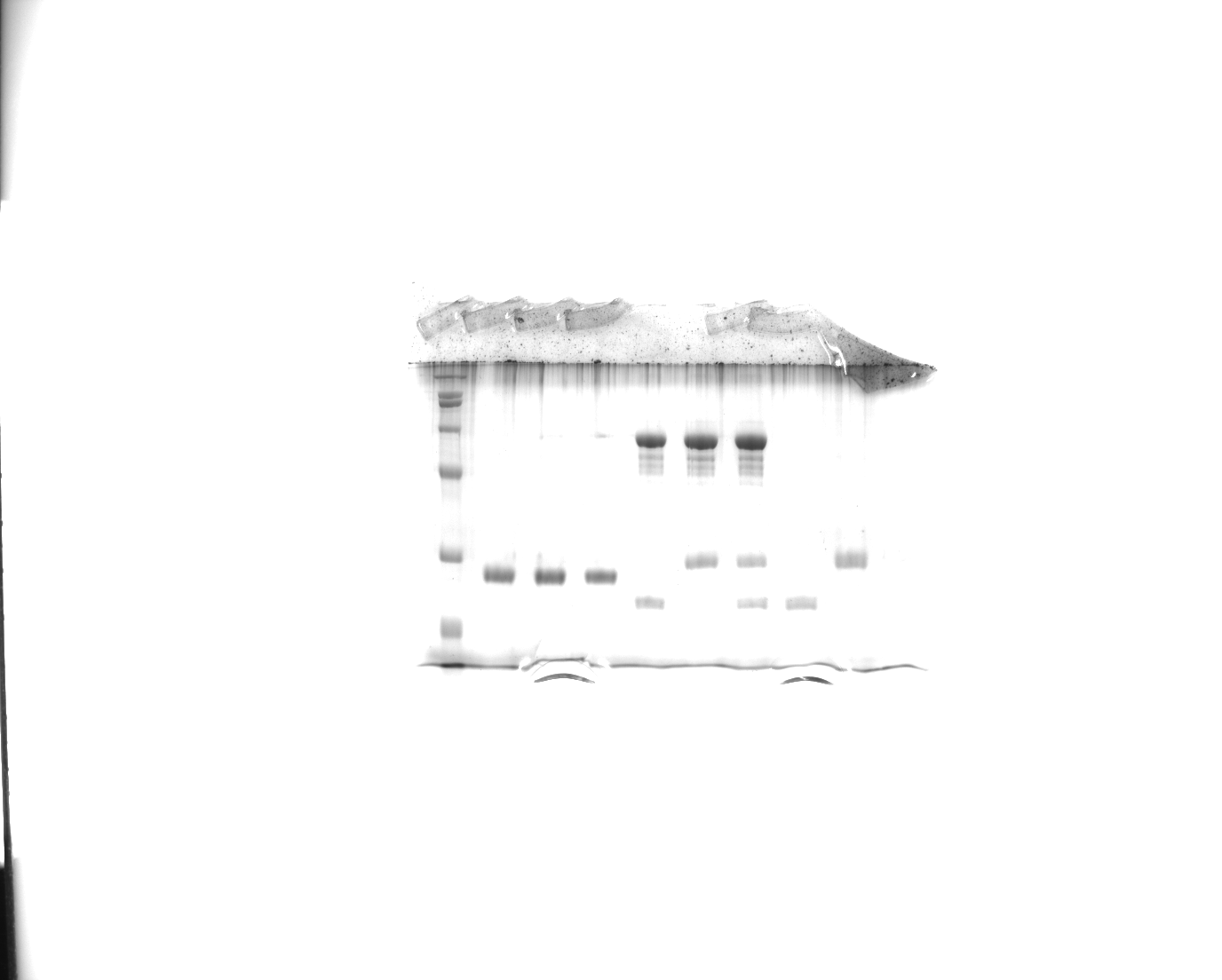

Supplement: Supplementary file 4 — Source data [file 41467_2023_37267_MOESM4_ESM.zip › source data/FIG2F.Tif]

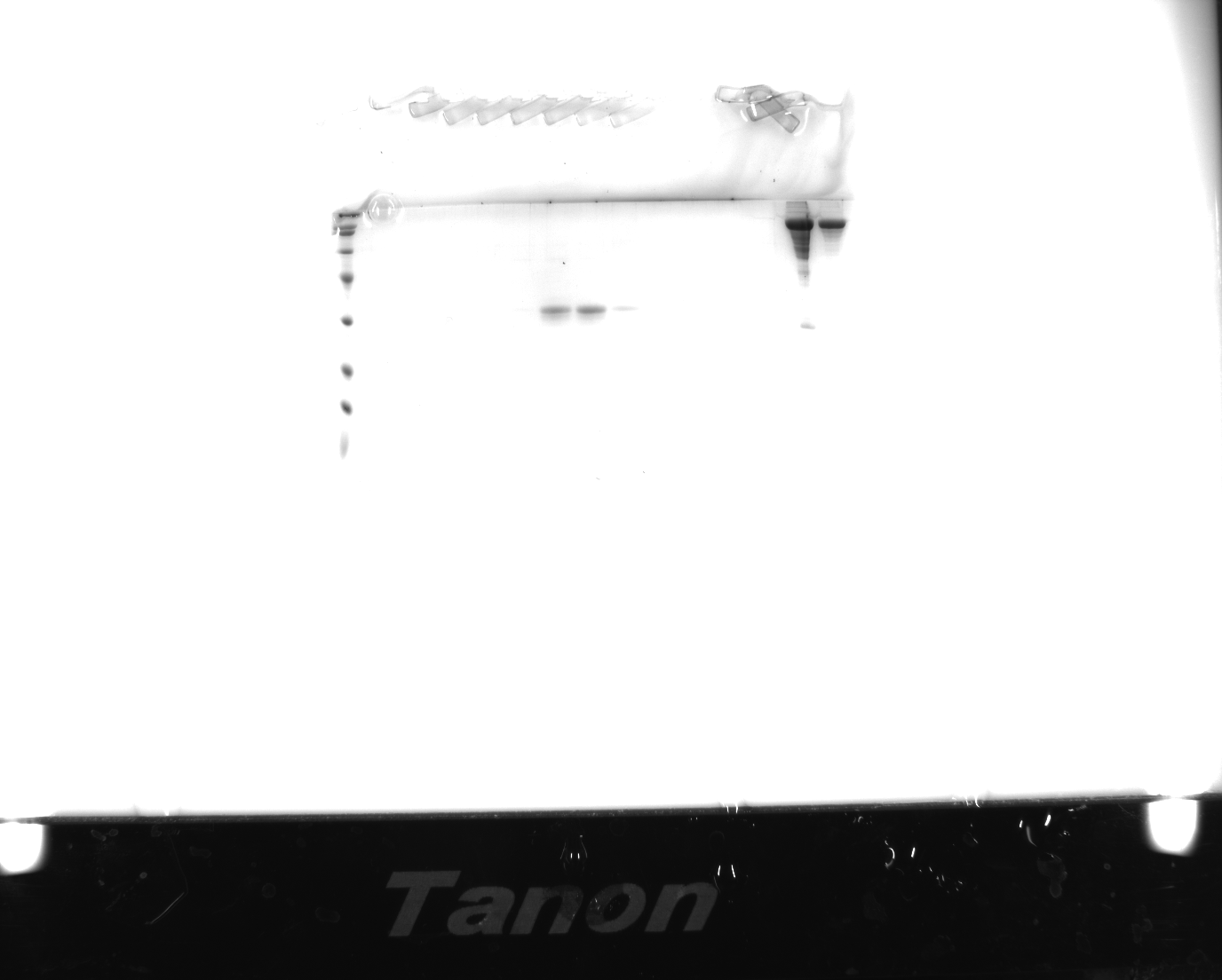

Supplement: Supplementary file 4 — Source data [file 41467_2023_37267_MOESM4_ESM.zip › source data/FIG3D ADGRV1-CT.jpg]

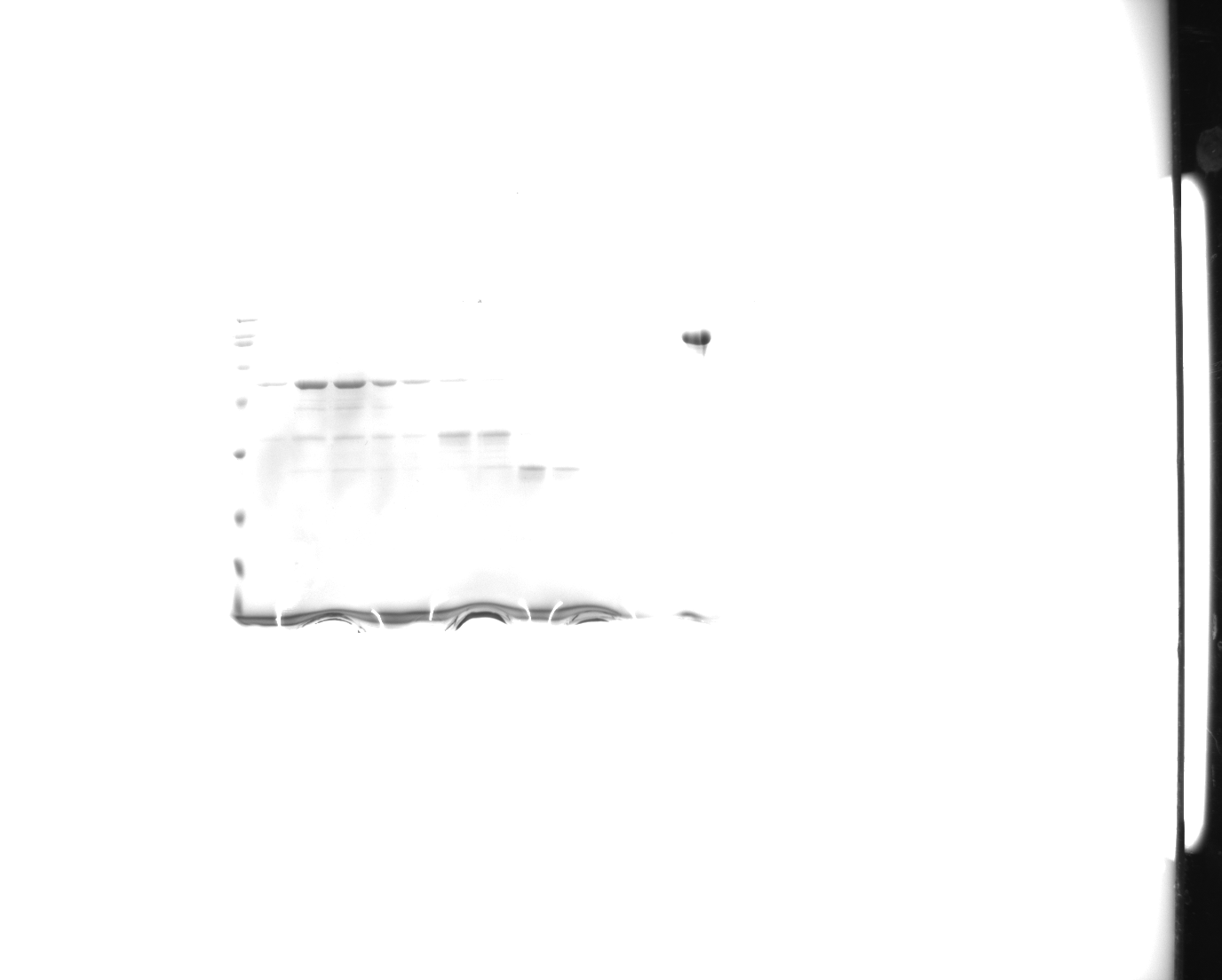

Supplement: Supplementary file 4 — Source data [file 41467_2023_37267_MOESM4_ESM.zip › source data/FIG3D Mixture.Tif]

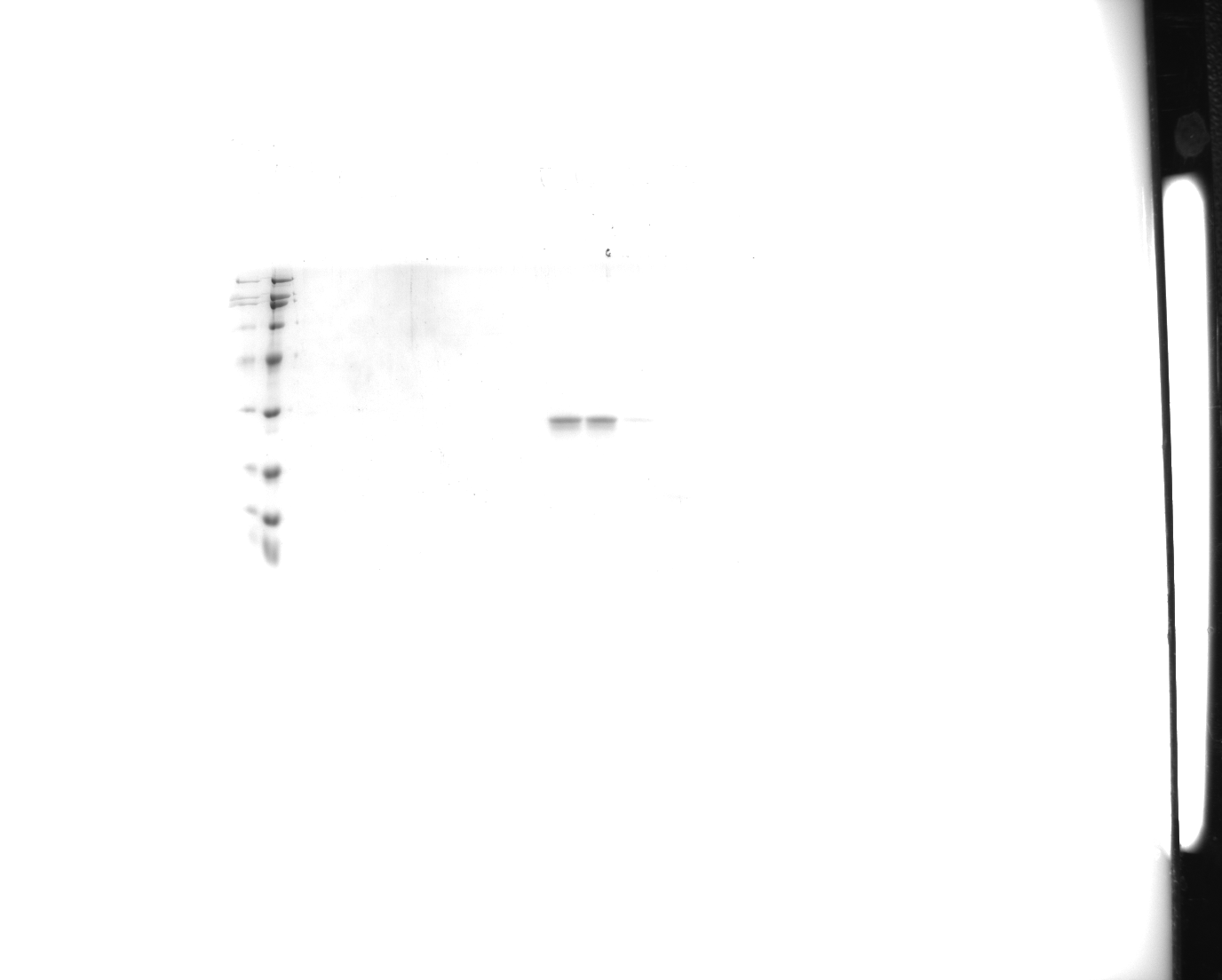

Supplement: Supplementary file 4 — Source data [file 41467_2023_37267_MOESM4_ESM.zip › source data/FIG3D USH2A-CT(S).Tif]

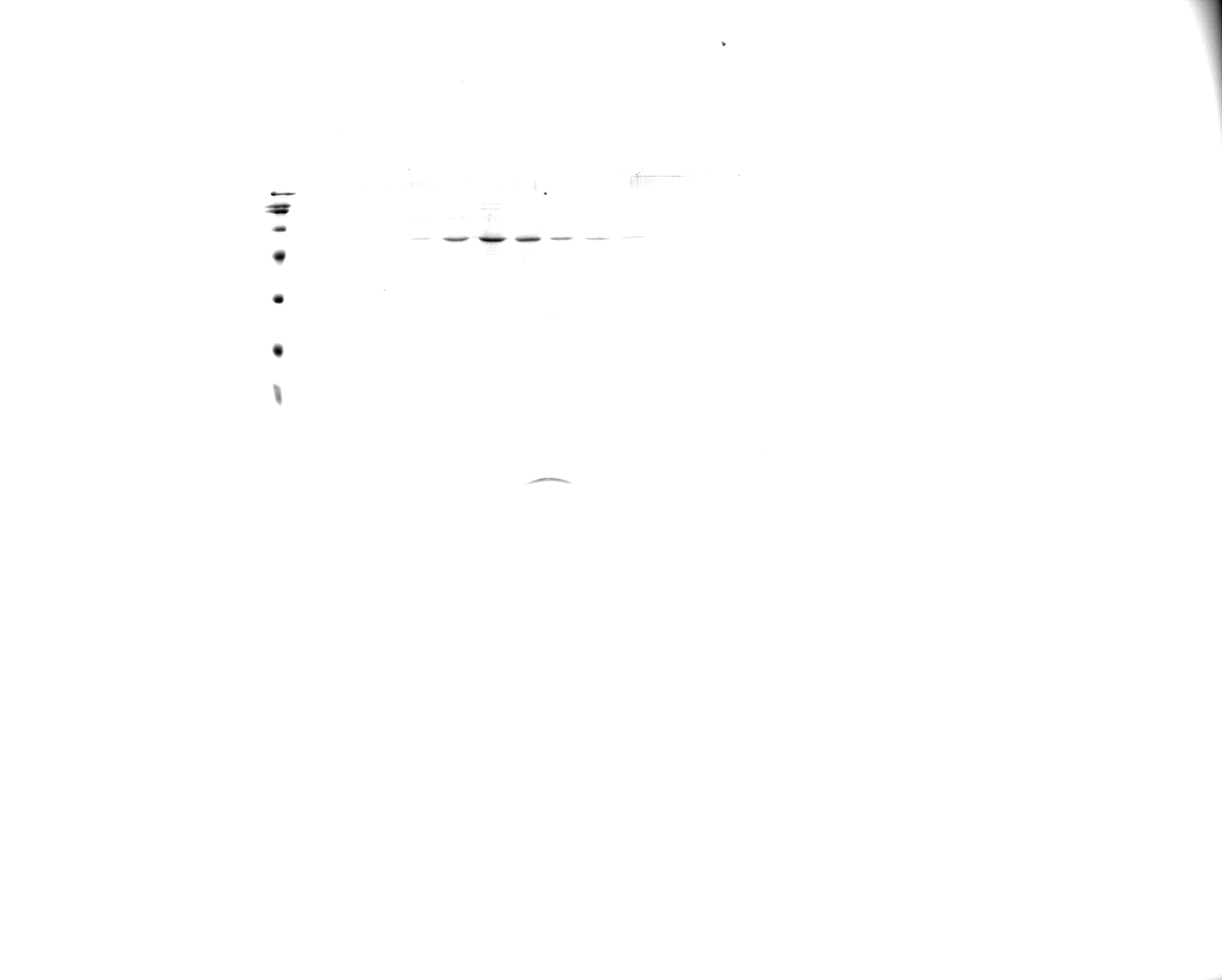

Supplement: Supplementary file 4 — Source data [file 41467_2023_37267_MOESM4_ESM.zip › source data/FIG3D WHRN.jpg]

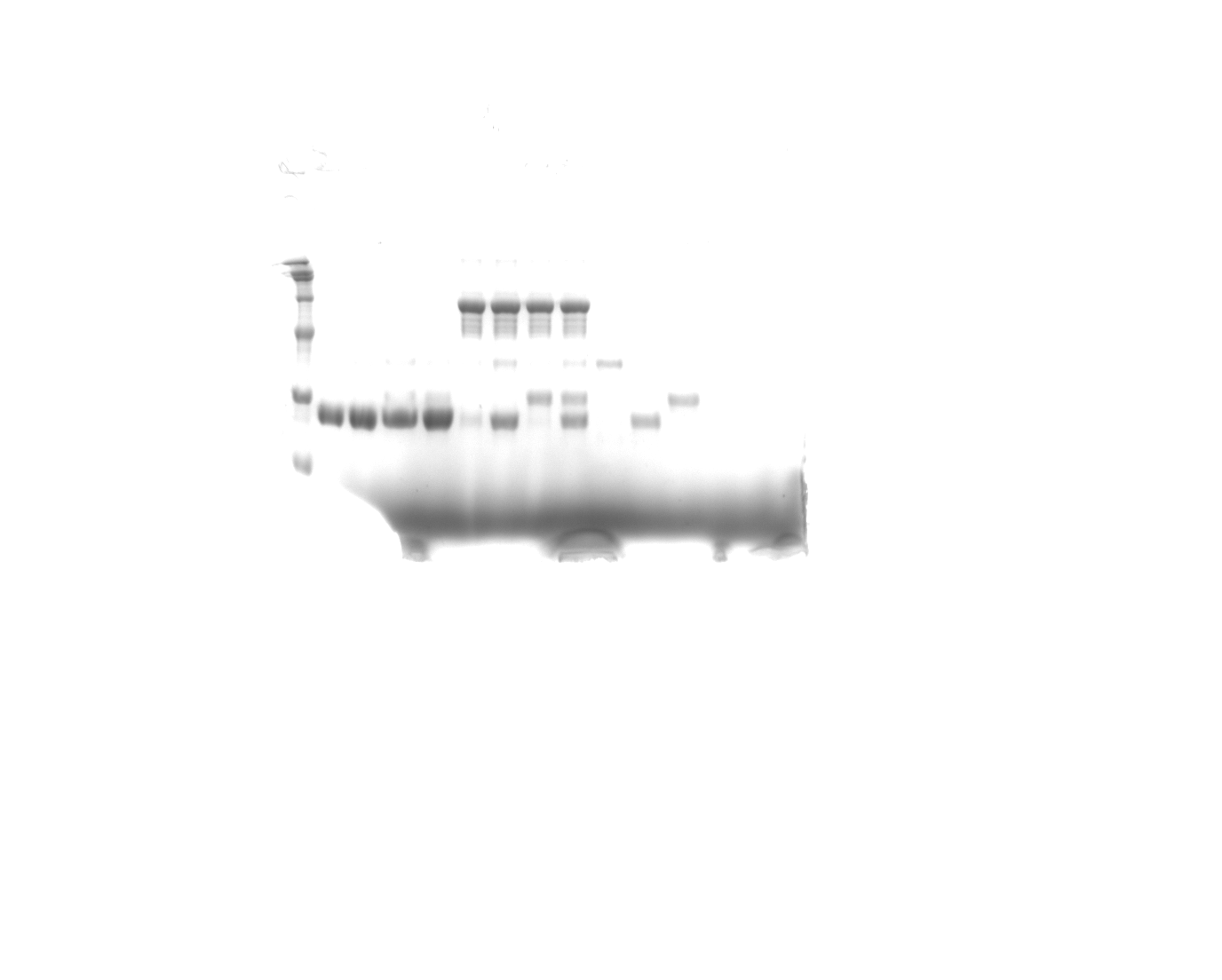

Supplement: Supplementary file 4 — Source data [file 41467_2023_37267_MOESM4_ESM.zip › source data/FIG4C.Tif]

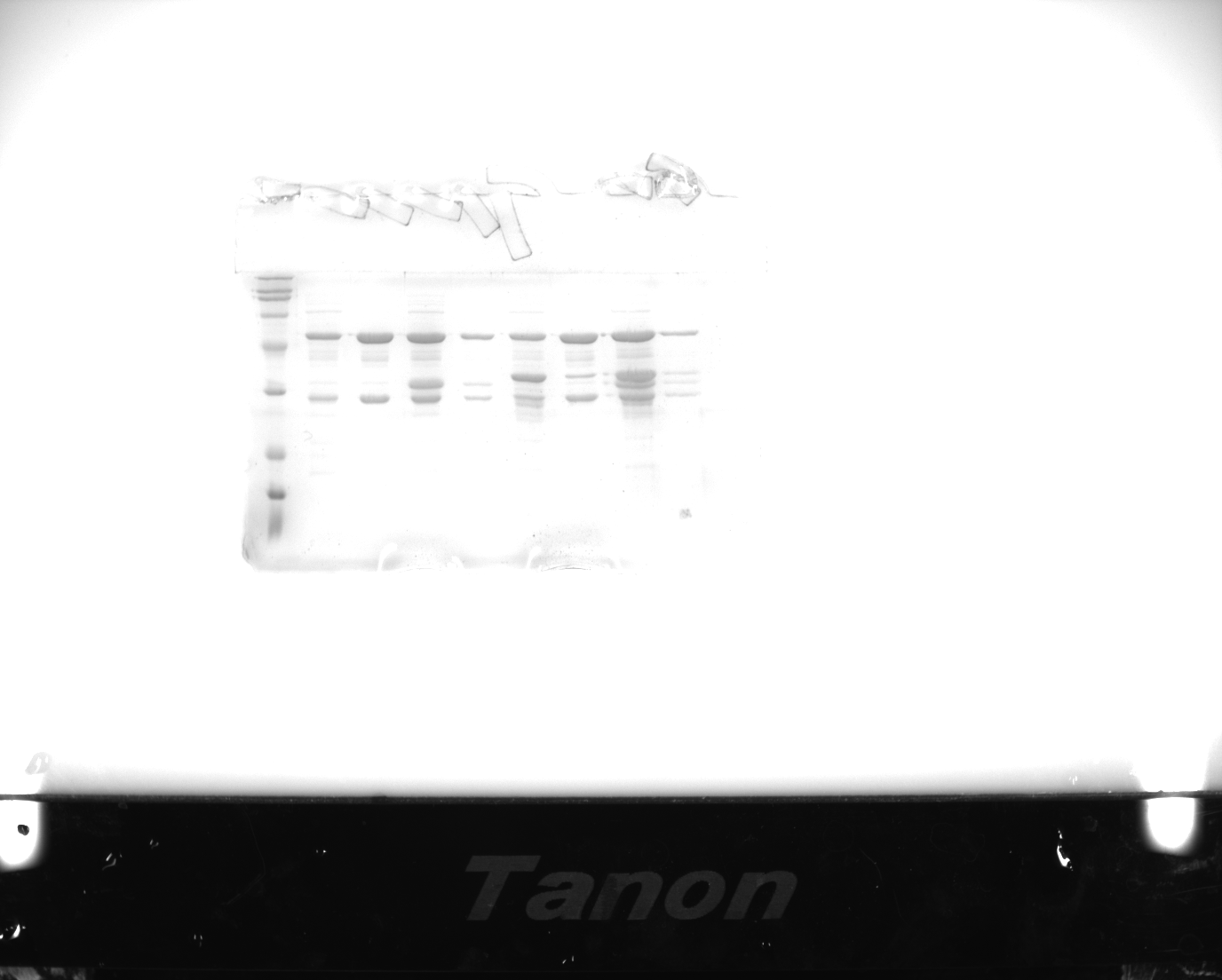

Supplement: Supplementary file 4 — Source data [file 41467_2023_37267_MOESM4_ESM.zip › source data/FIG4D.Tif]

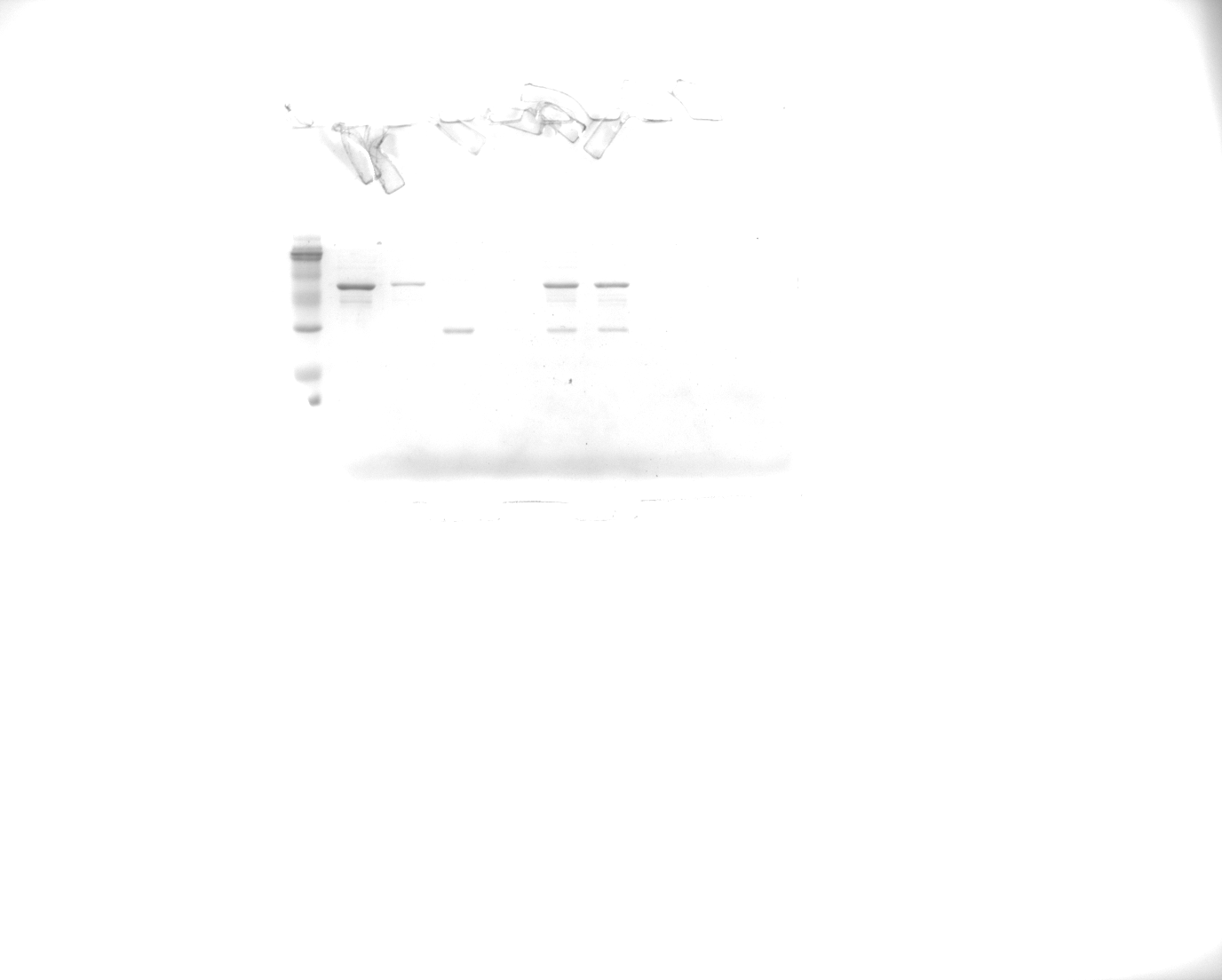

Supplement: Supplementary file 4 — Source data [file 41467_2023_37267_MOESM4_ESM.zip › source data/FIG5A.Tif]

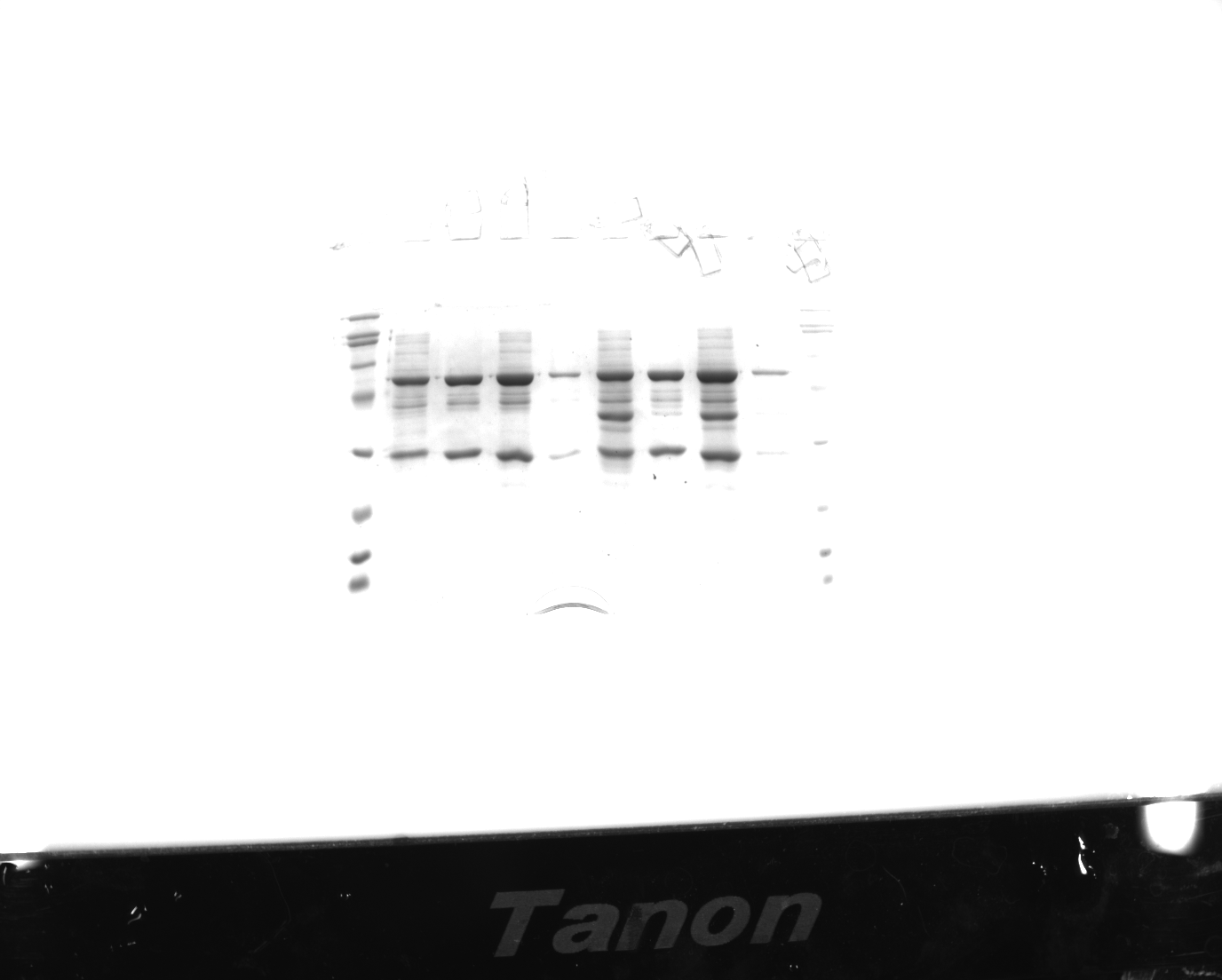

Supplement: Supplementary file 4 — Source data [file 41467_2023_37267_MOESM4_ESM.zip › source data/FIG5D.Tif]

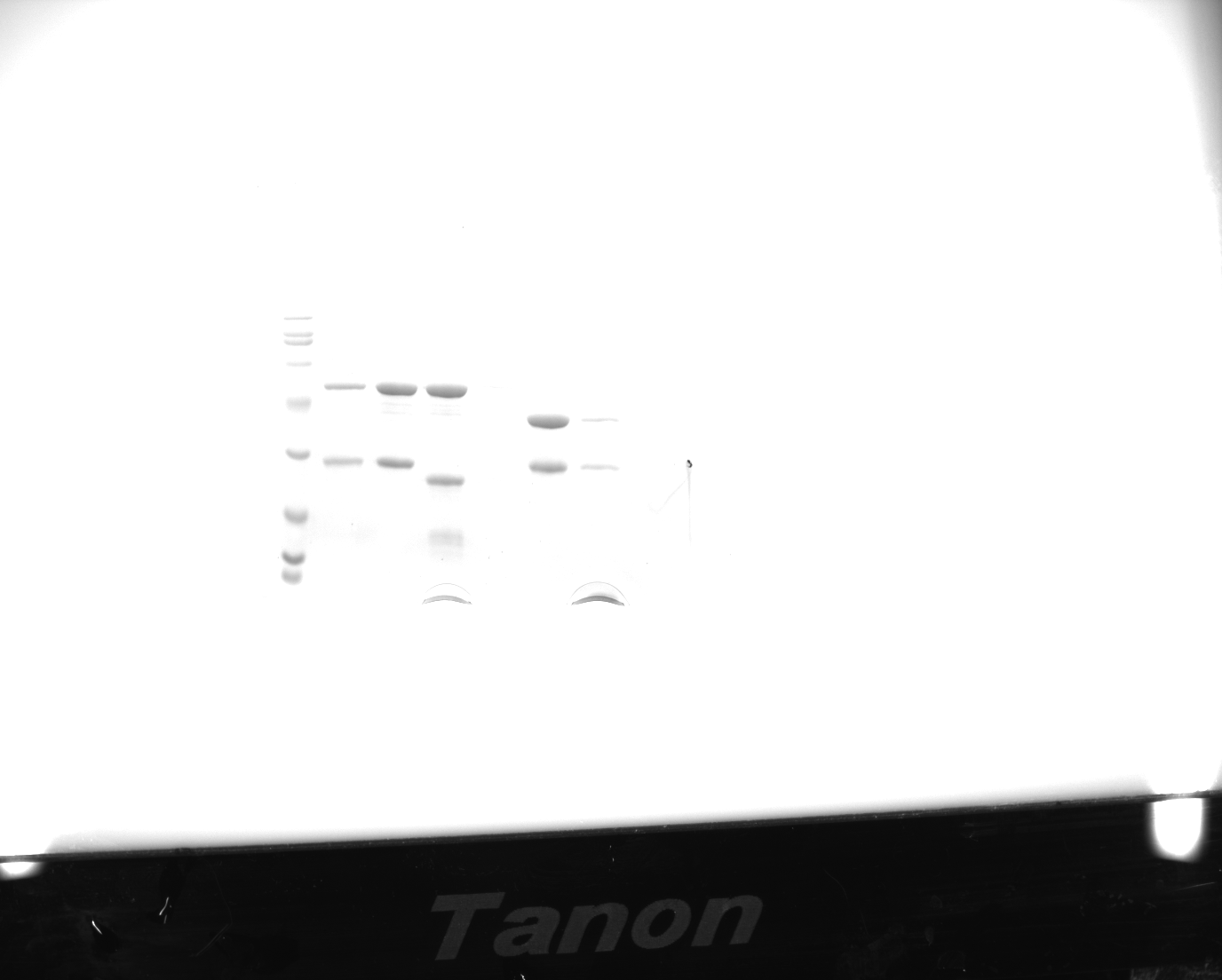

Supplement: Supplementary file 4 — Source data [file 41467_2023_37267_MOESM4_ESM.zip › source data/FIG5E.Tif]

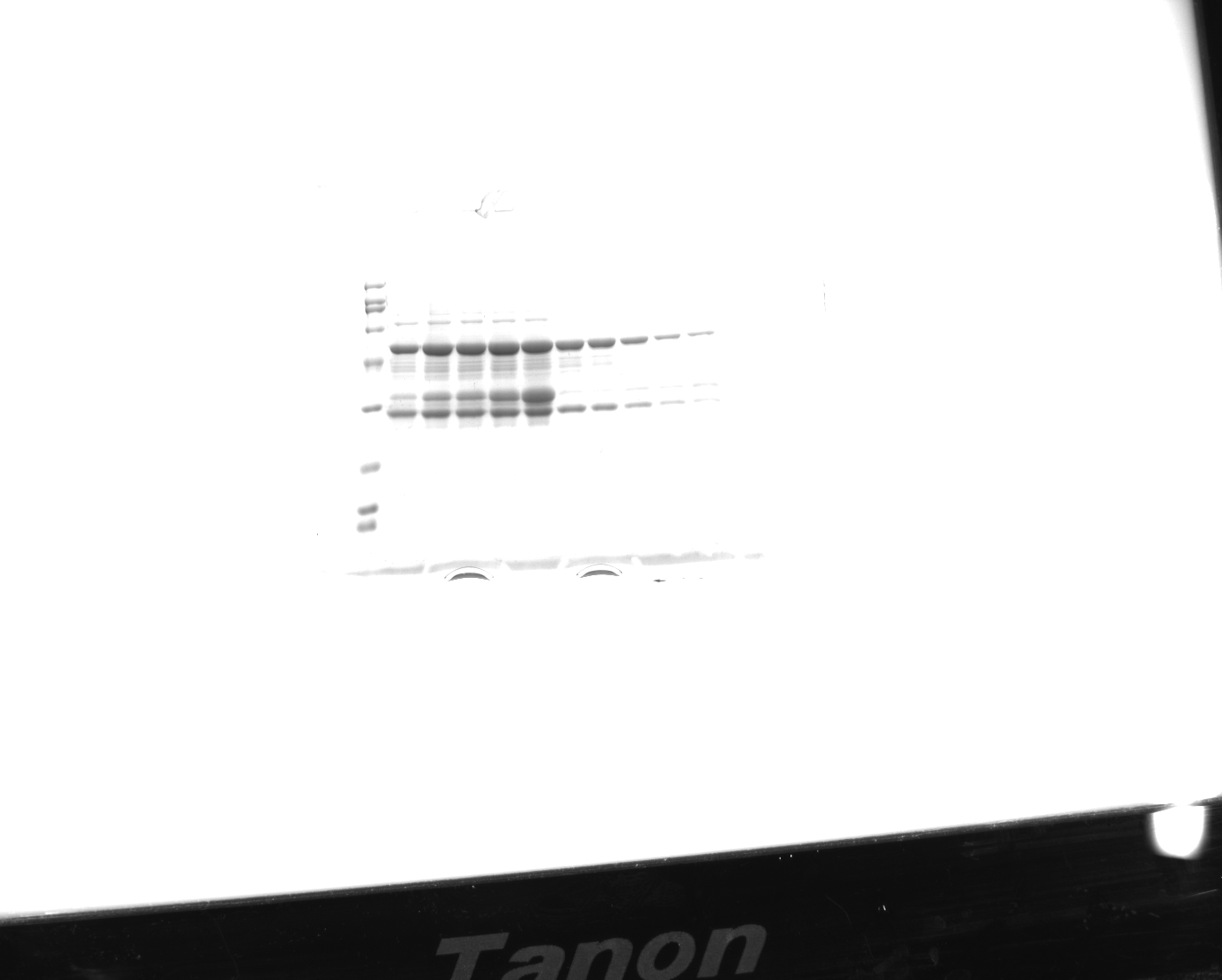

Supplement: Supplementary file 4 — Source data [file 41467_2023_37267_MOESM4_ESM.zip › source data/FIG6A.Tif]

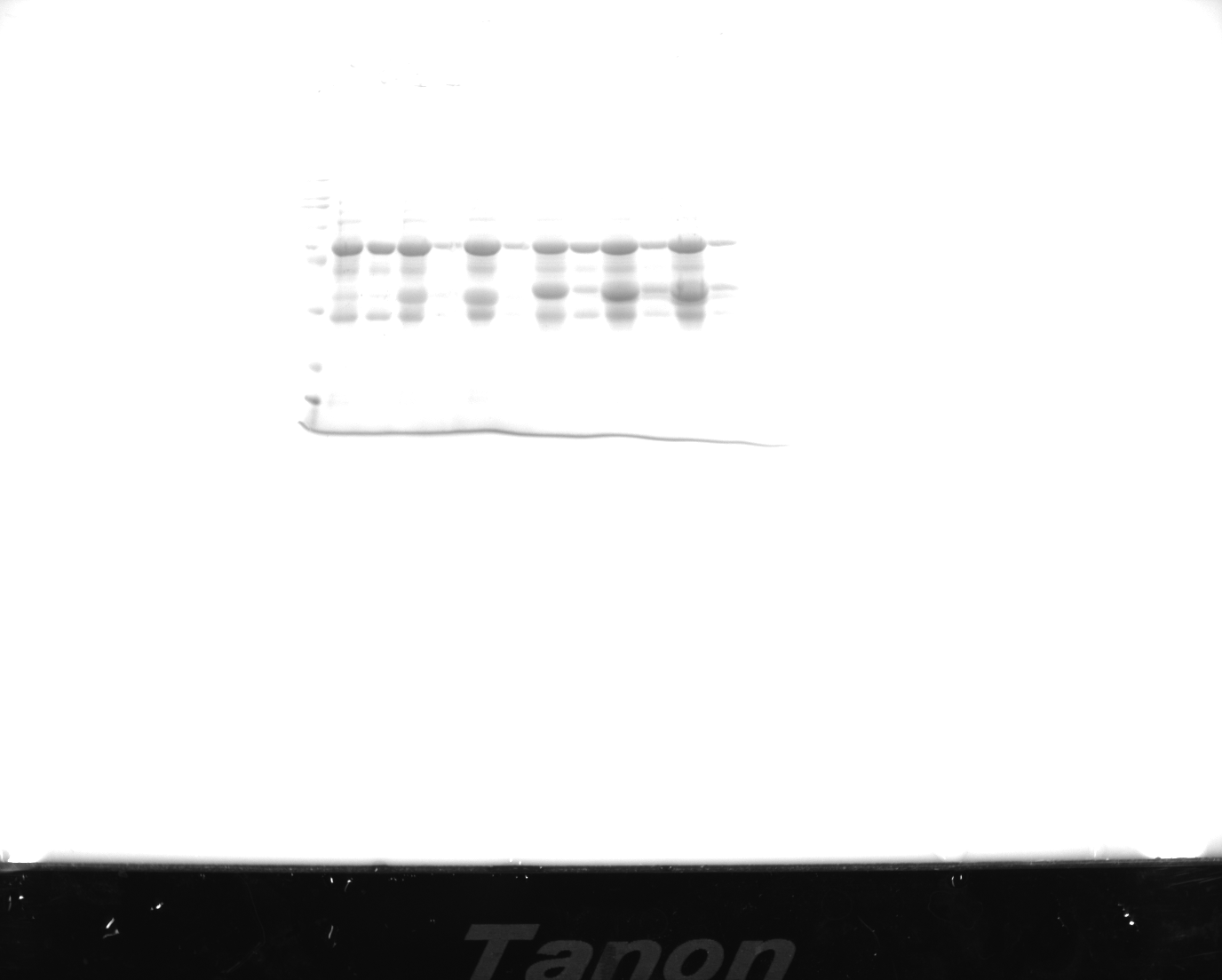

Supplement: Supplementary file 4 — Source data [file 41467_2023_37267_MOESM4_ESM.zip › source data/FIG6C.Tif]

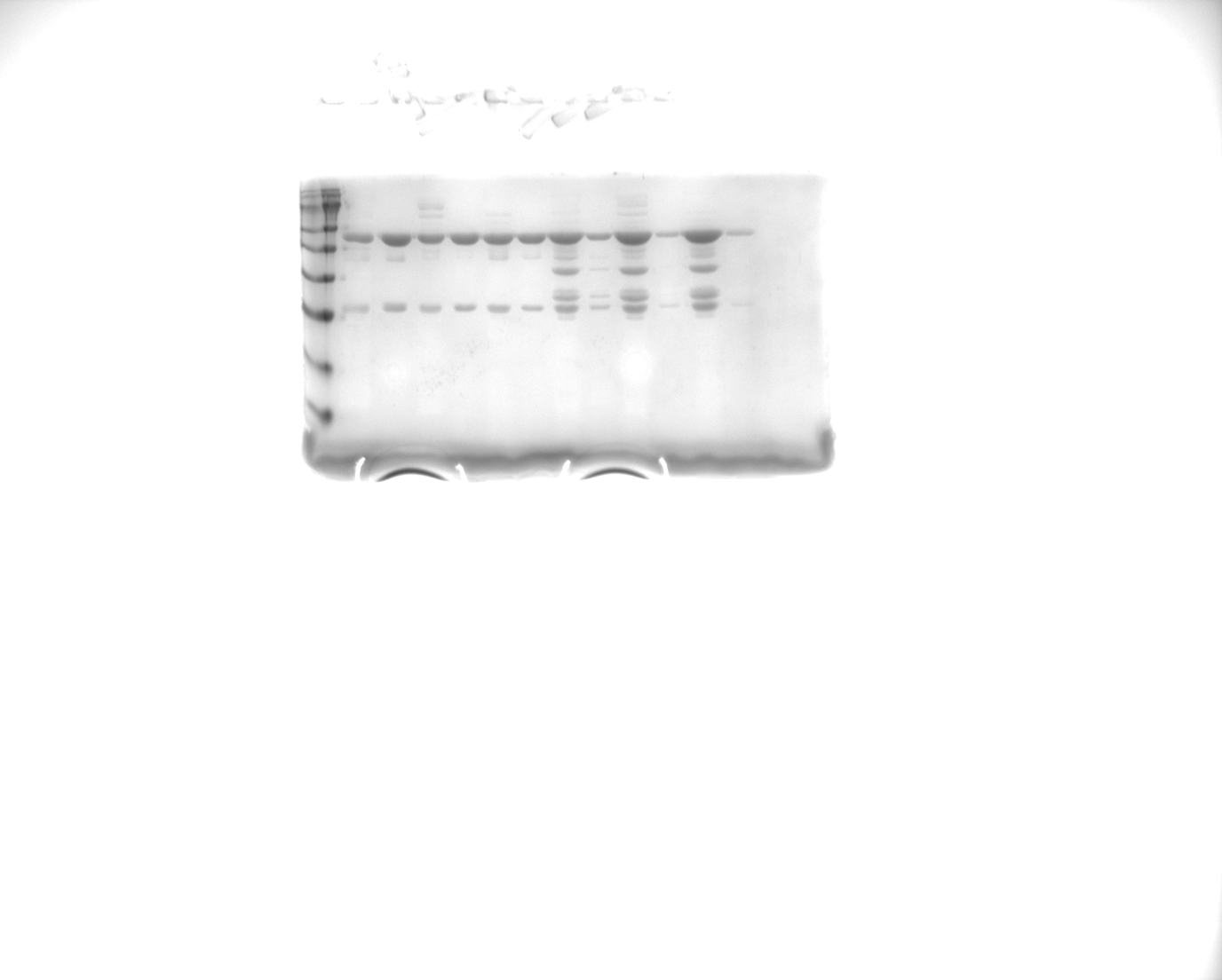

Supplement: Supplementary file 4 — Source data [file 41467_2023_37267_MOESM4_ESM.zip › source data/FIG8E.Tif]

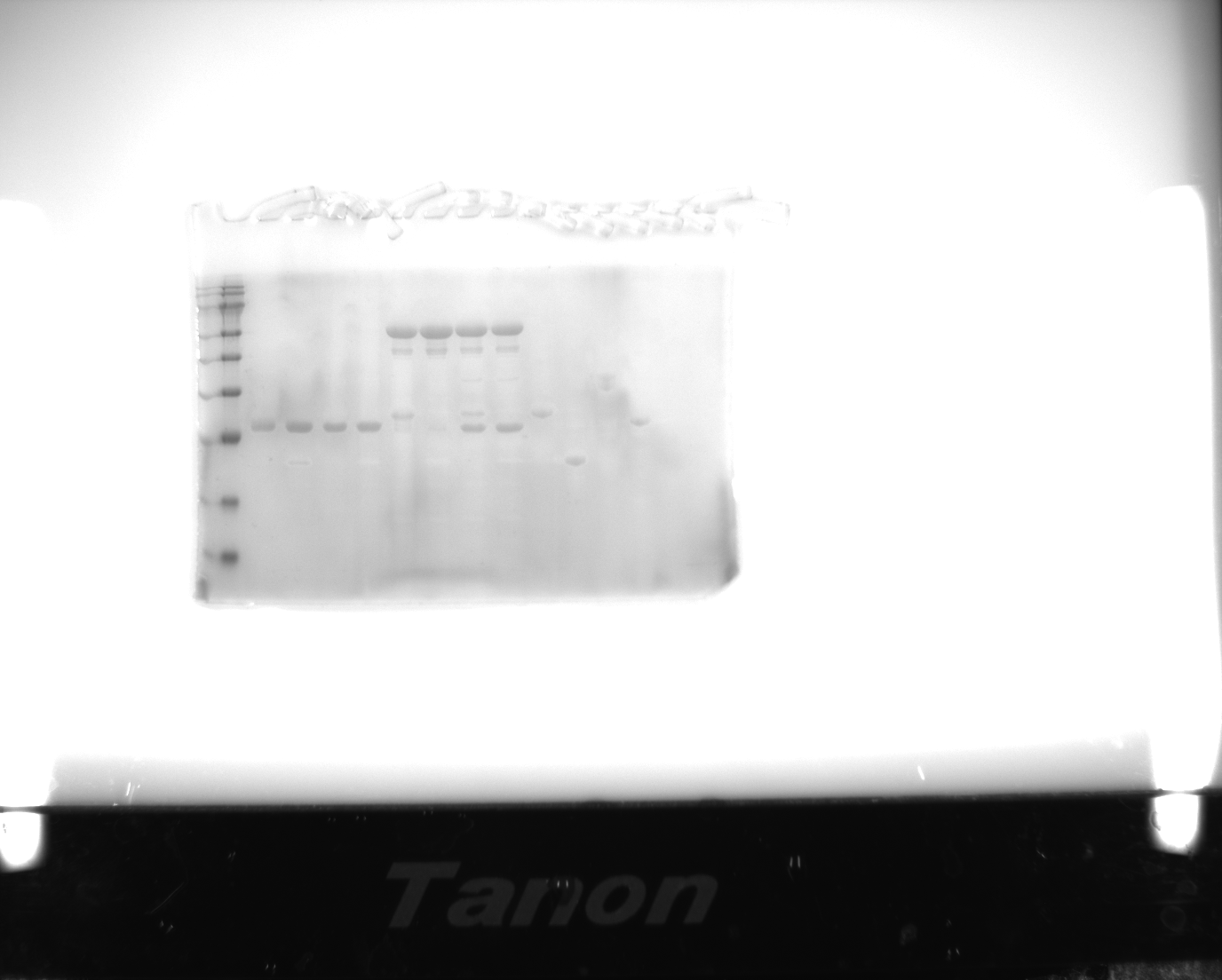

Supplement: Supplementary file 4 — Source data [file 41467_2023_37267_MOESM4_ESM.zip › source data/FIG9A.Tif]

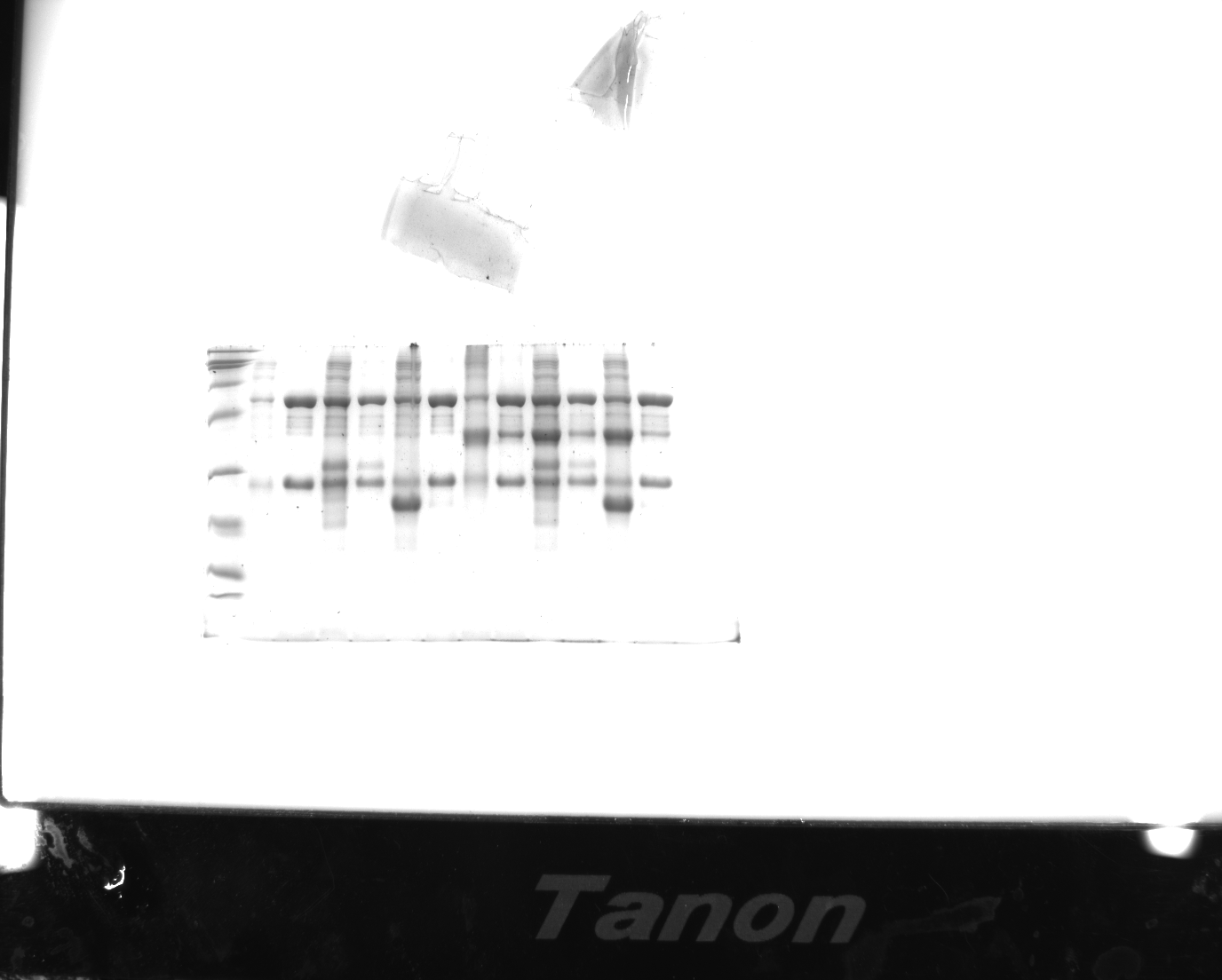

Supplement: Supplementary file 4 — Source data [file 41467_2023_37267_MOESM4_ESM.zip › source data/FIG9B.Tif]

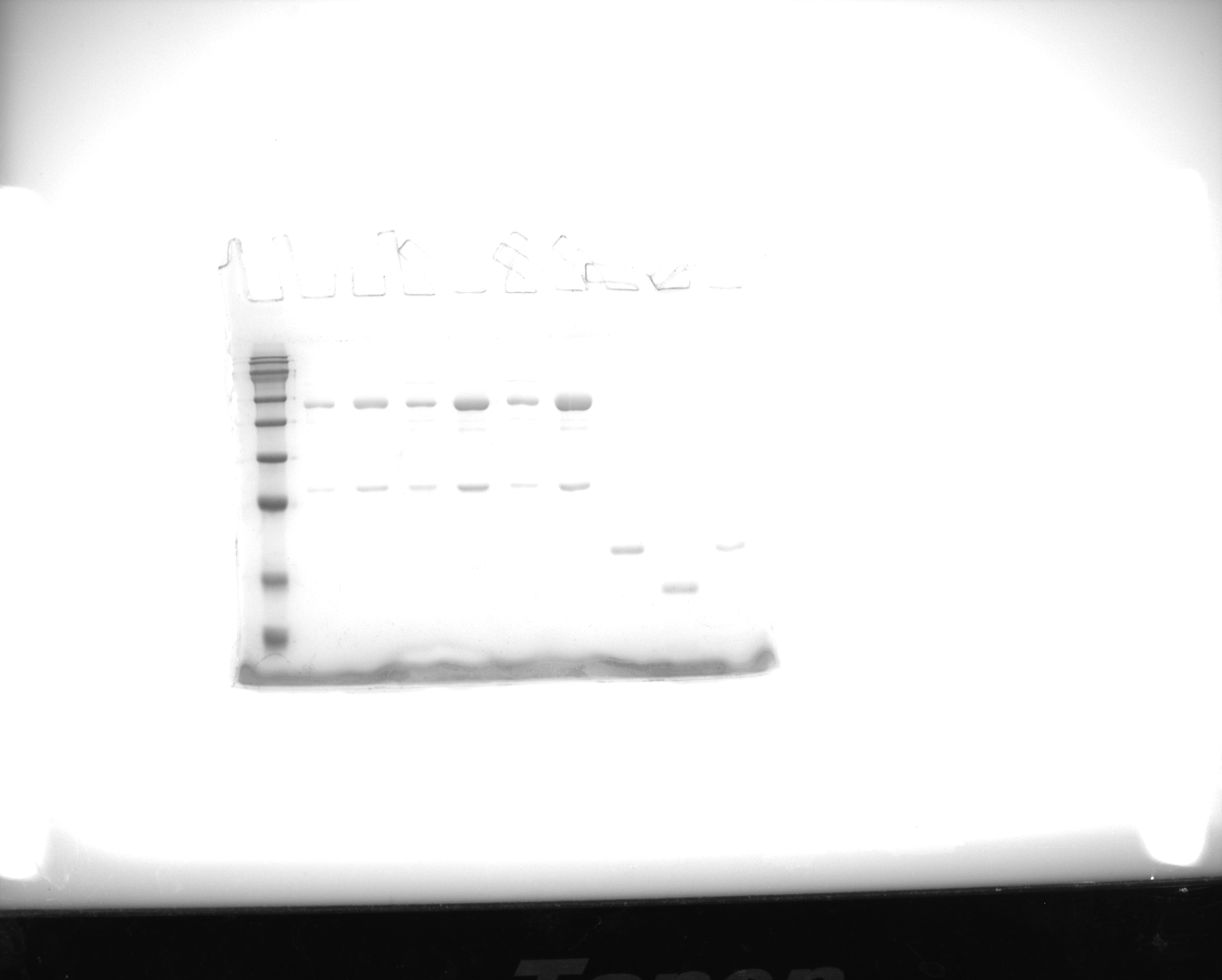

Supplement: Supplementary file 4 — Source data [file 41467_2023_37267_MOESM4_ESM.zip › source data/Supplementary FIG5A.Tif]

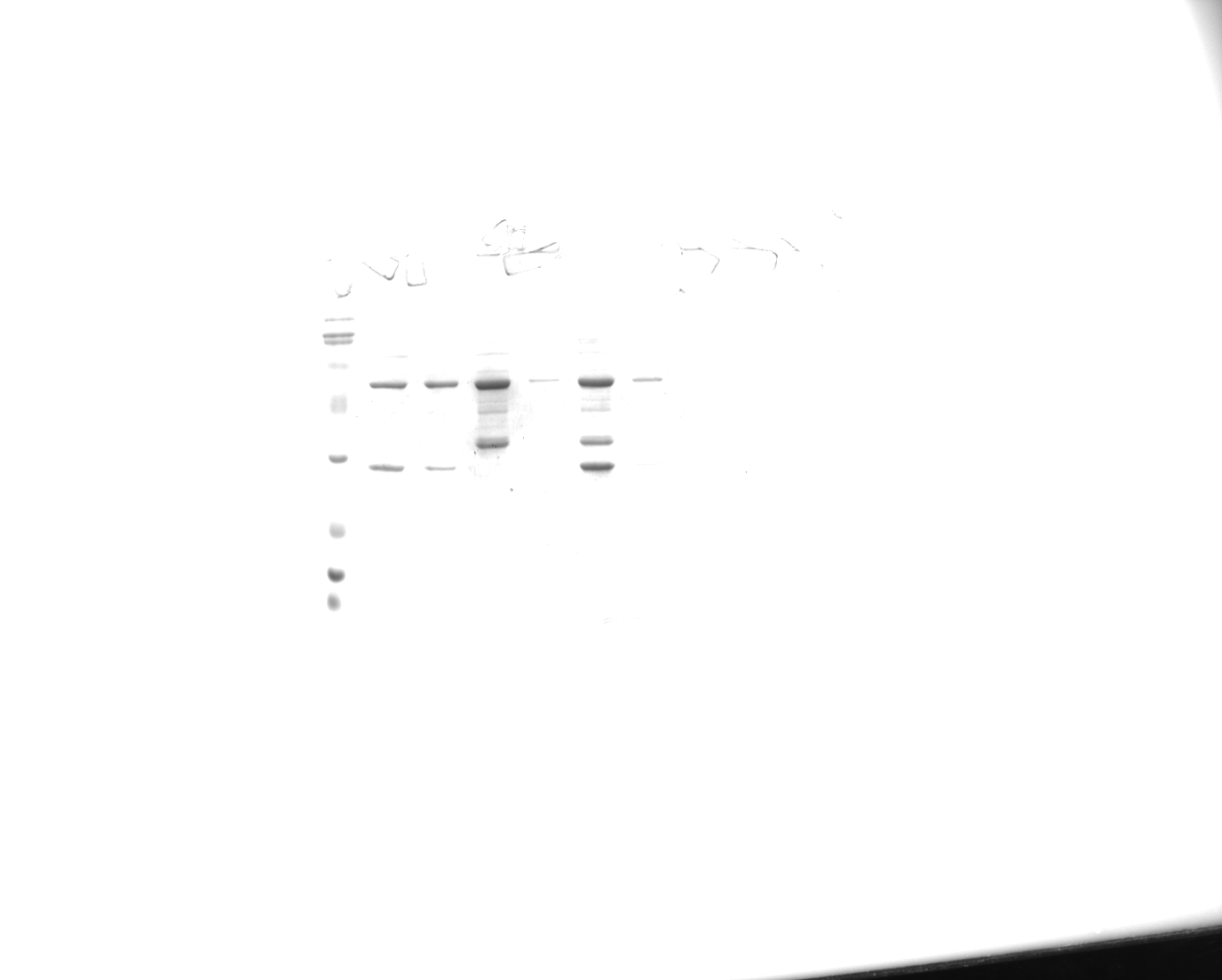

Supplement: Supplementary file 4 — Source data [file 41467_2023_37267_MOESM4_ESM.zip › source data/Supplementary FIG6A.Tif]
